# Supplementary figures and images for: Closed-domain event extraction for hard news event monitoring: a systematic study
Source: PeerJ Comput Sci. 2024 Oct 10;10:e2355. doi: 10.7717/peerj-cs.2355 (PMC11623237; doi:10.7717/peerj-cs.2355)

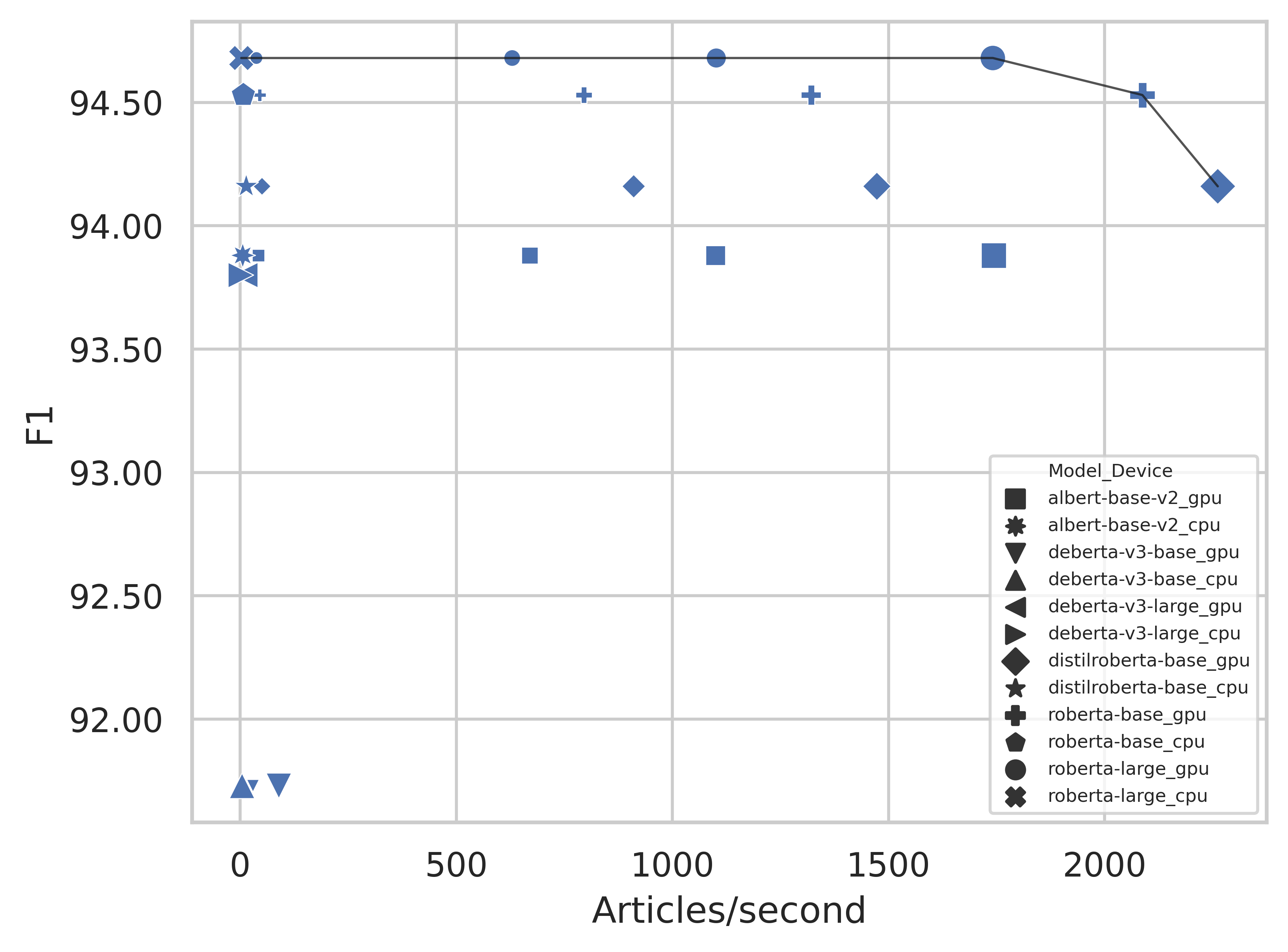

Supplement: Supplemental Information 1 [file peerj-cs-10-2355-s001.zip › evaluate_hardware/figure_4a.png]

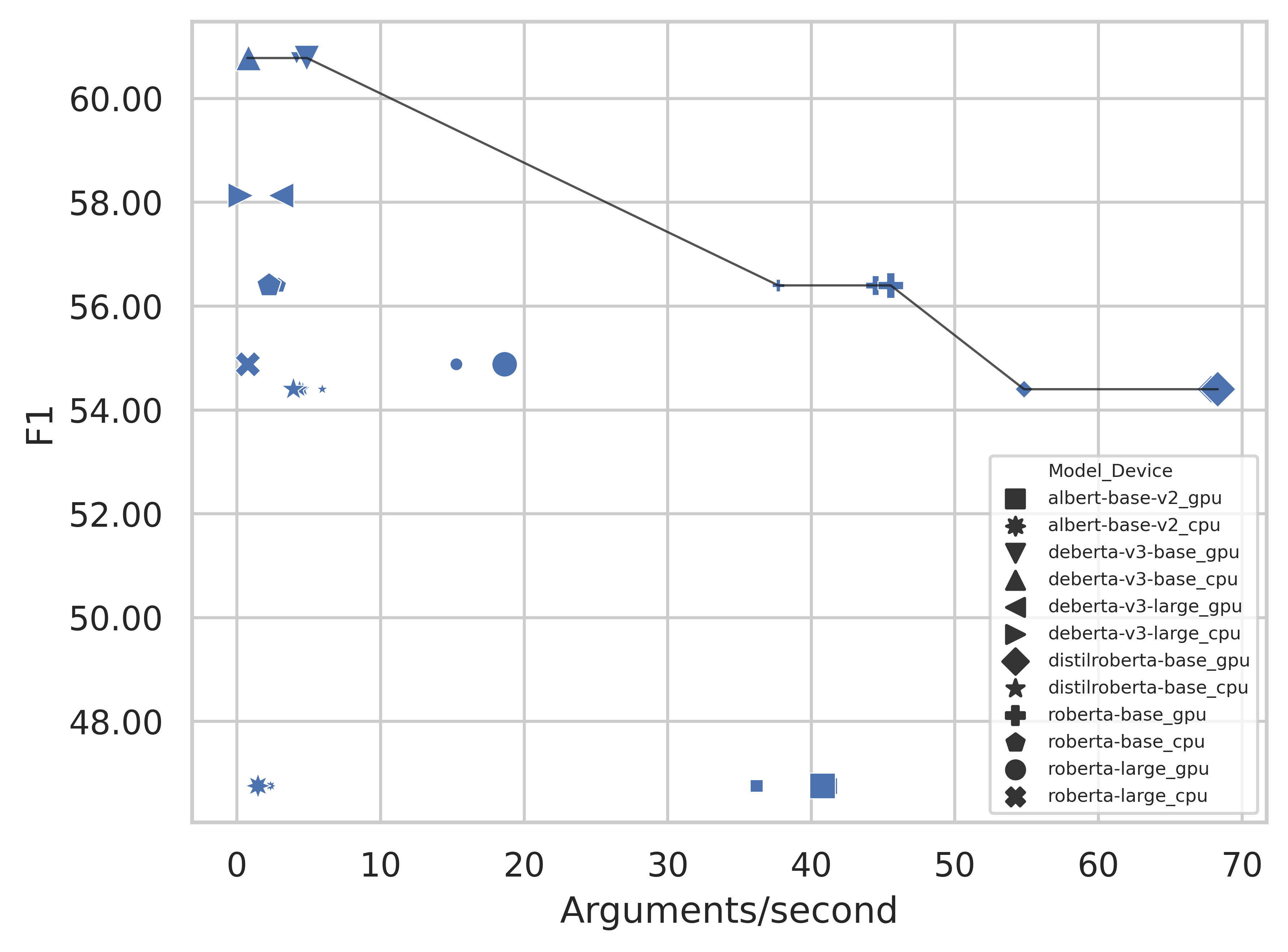

Supplement: Supplemental Information 1 [file peerj-cs-10-2355-s001.zip › evaluate_hardware/figure_4b.png]

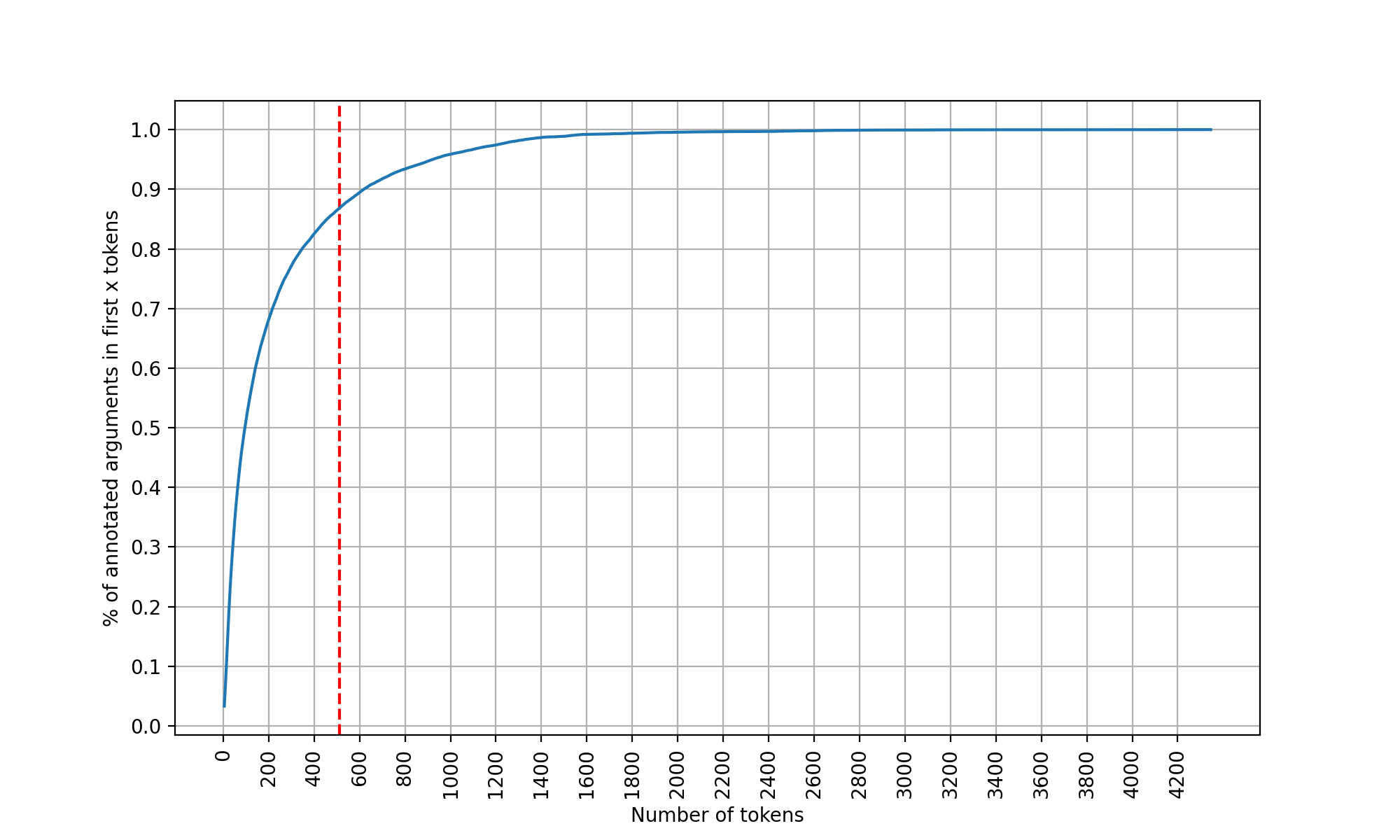

Supplement: Supplemental Information 1 [file peerj-cs-10-2355-s001.zip › notebooks/arg_ext_seq_lab_hn/test_arg_percentage.png]

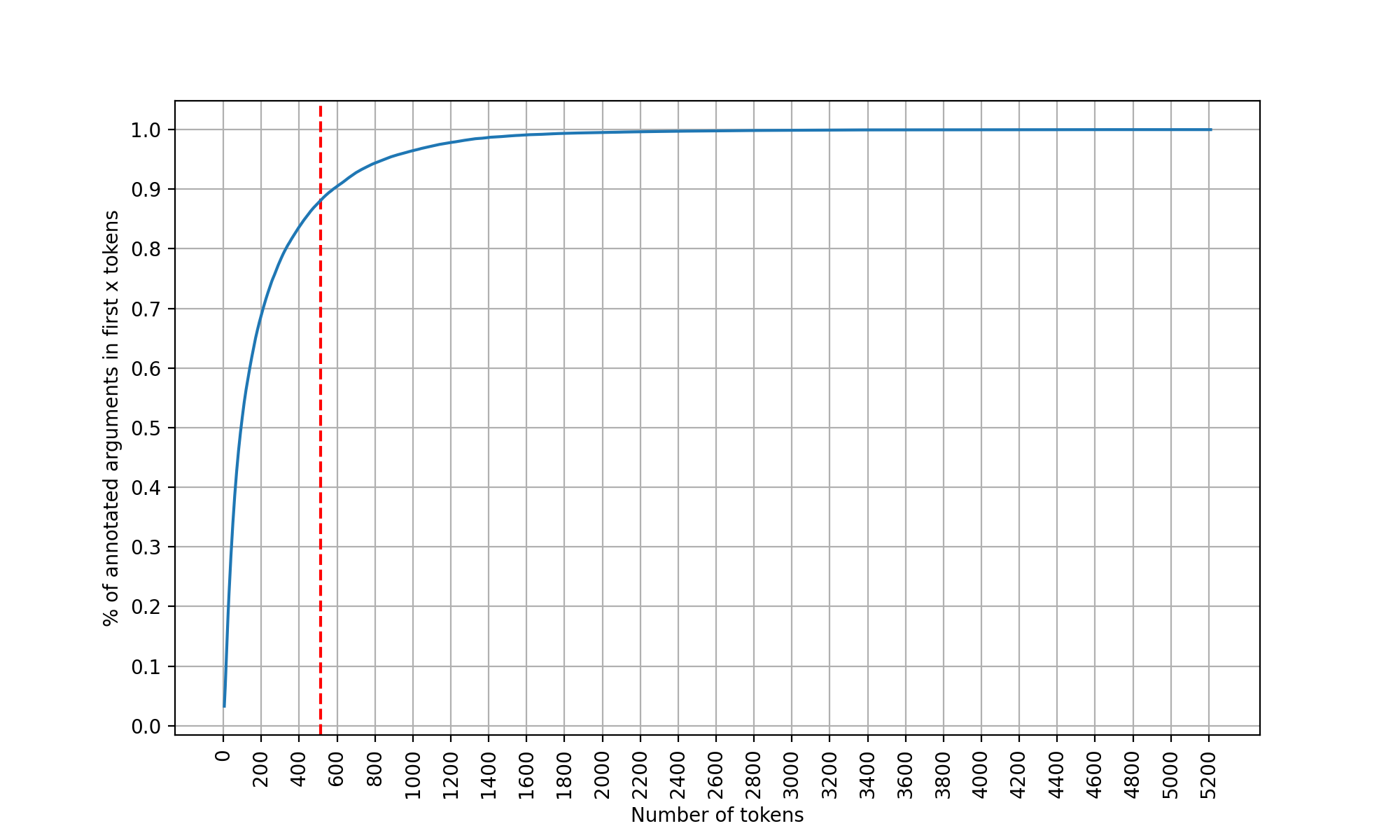

Supplement: Supplemental Information 1 [file peerj-cs-10-2355-s001.zip › notebooks/arg_ext_seq_lab_hn/train_arg_percentage.png]
